# Minimizing Manual Image Segmentation Turn-Around Time for Neuronal Reconstruction by Embracing Uncertainty

**DOI:** 10.1371/journal.pone.0044448

**Published:** 2012-09-21

**Authors:** Stephen M. Plaza, Louis K. Scheffer, Mathew Saunders

**Affiliations:** Janelia Farm Research Campus, Ashburn, Virginia, United States of America; University of Iowa, United States of America

## Abstract

The ability to automatically segment an image into distinct regions is a critical aspect in many visual processing applications. Because inaccuracies often exist in automatic segmentation, manual segmentation is necessary in some application domains to correct mistakes, such as required in the reconstruction of neuronal processes from microscopic images. The goal of the automated segmentation tool is traditionally to produce the highest-quality segmentation, where quality is measured by the similarity to actual ground truth, so as to minimize the volume of manual correction necessary. Manual correction is generally orders-of-magnitude more time consuming than automated segmentation, often making handling large images intractable. Therefore, we propose a more relevant goal: minimizing the turn-around time of automated/manual segmentation while attaining a level of similarity with ground truth. It is not always necessary to inspect every aspect of an image to generate a useful segmentation. As such, we propose a strategy to guide manual segmentation to the most uncertain parts of segmentation. Our contributions include 1) a probabilistic measure that evaluates segmentation without ground truth and 2) a methodology that leverages these probabilistic measures to significantly reduce manual correction while maintaining segmentation quality.

## Introduction

The proper segmentation of an image can facilitate analysis useful in many applications. In this paper, we will focus on the reconstruction of neural connectivity from electronic microscope (EM) images as the primary driver application for our approach. To discern the neural connectivity, an EM image is segmented into different bodies and those portions are linked to each other in a manner that is consistent with boundaries visible in the image to form reconstructed neurites. Because of the large volume of image data, tens of thousands of images are typical, automatic segmentation is employed. However, because small errors in the segmentation can result in large topological errors, manual inspection of the entire volume is necessary to correct any errors [1].

Manual segmentation poses many difficulties. First, segmentation itself is an inexact operation. Conceptually, there are often many ways to segment an image that similarly trained experts may disagree upon. Because of this, there is an inherent ambiguity in even the so-called, ground-truth segmentation. The authors in [2], [3] describe a similarity metric that incorporates this uncertainty among different ground truths. Second, results rely on the inspection of the entire image volume, even if automatic segmentation is used as the starting point. This need to look at every pixel forms a lower bound on the reconstruction effort, even if no errors are discovered or corrected.

In this paper, we consider the goal of segmentation as one that simultaneously produces something close to ground truth while minimizing the time for manual verification. Typically, manual verification turn-around time is characterized as a function of errors needed to be corrected, i.e., nuisance metric. However, we introduce a different formulation of the work required in segmentation to account for other factors such as the percentage of image volume that needs to be examined. In this manner, we explicitly formulate a goal metric allowing a graceful tradeoff between the quality of the segmentation and the amount of work required to manually verify/correct the segmentation. In general, many applications do not require 100% accuracy. In [1], the dense reconstruction of neural connectivity facilitates the classification of cell types and ease of synaptic tracing. Small topological errors in this domain will likely not interfere with either objective and in practice many such errors are explicitly accepted in past methodology.

To facilitate such a system that trades off accuracy and turn-around time, we propose several contributions. First, we introduce a segmentation methodology that decomposes the problem of image segmentation to a series of graph manipulations where each node represents a set of image voxels and the edge strength represents the connectivity between nodes [4], [5]. We refer to such a graph as a *probabilistic segmentation graph*. The system shares conceptual similarities to that proposed in [4] with the important difference that the edge strengths are not reduced to yes/no decisions. The connectivity rather indicates the probabilistic certainty of nodes being connected.

**Figure 1:**
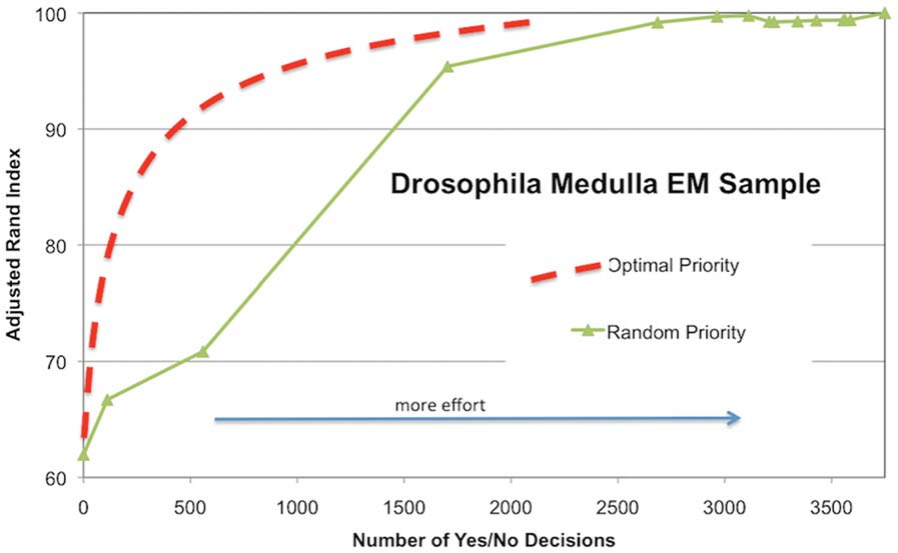
Tradeoff between effort and quality of segmentation. The quality of reconstruction is shown as a function of the reconstruction effort. Randomly finding places to verify leads to slow improvements in segmentation quality.

We introduce similarity metrics to evaluate the quality of the segmentation without the presence of ground truth. Typically, ground truth is useful to evaluate the effectiveness of a segmentation algorithm. Because a priori ground truth is not generally available for real applications, we introduce a novel self-referential similarity metric that uses the probabilistic segmentation graph as its primary input. In this manner, we provide a number that shows how certain we are that the segmentation is correct. This is used as a guide for manual correction so that uncertain parts of the segmentation are examined first. More importantly, when enough parts are examined this certainty reaches a level where the manual correction can be safely terminated. We show results where more than 98% of manual correction is avoided while maintaining an acceptable level of accuracy. This motivation is illustrated in Figure 1 that shows how the quality of segmentation varies as a function of manual effort (image taken from the *Drosophila*/fly Medulla). When manual effort is not focused on the most important aspect of the image, segmentation improvements occur slowly. We introduce a prioritization that behaves more closely to the ideal scenario of achieving the most improvement with the smallest amount of work.

We first discuss previous efforts in automatic segmentation, similarity metrics, and some background in our target application of neuronal reconstruction. Then we introduce our optimization objective and probabilistic graph. Next, we propose probabilistic measures for assessing the quality of this segmentation and ways to use these measures to guide manual correction. Finally, we present results that indicate the ability of our approach to reduce the time of manual correction.

## Background

This section briefly surveys the relevant segmentation strategies that we leverage in this work. In addition, we explain how the quality of the segmentation is assessed using different similarity metrics. Finally, this section explains the domain considered in our experiments: neuronal reconstruction.

### 1 Hierarchical Image Segmentation and Analysis

**Figure 2:**
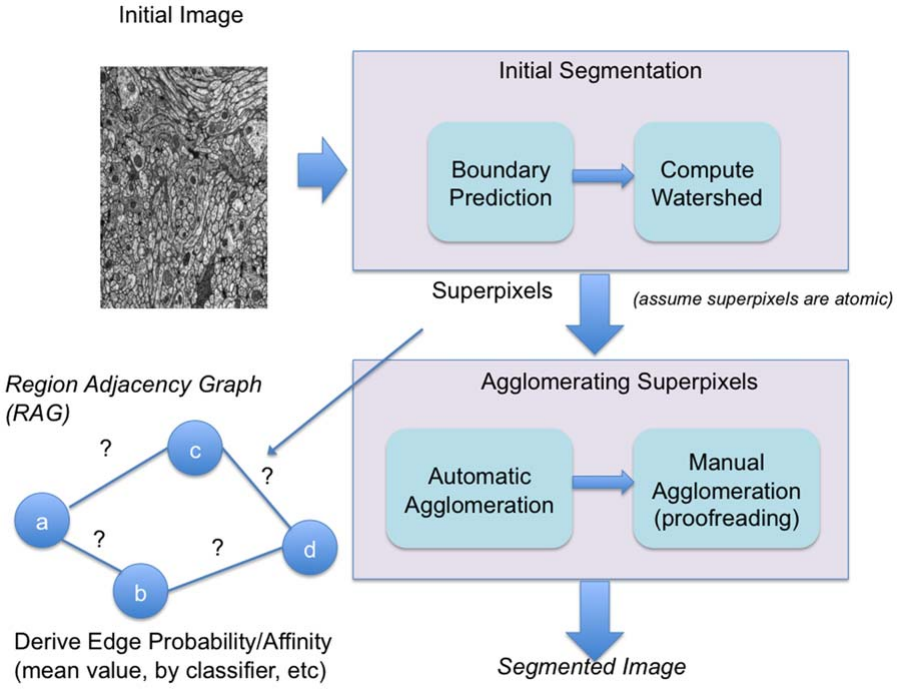
Segmentation workflow. The workflow consists of boundary prediction and agglomeration including automatic and manual effort.

The goal of image segmentation as shown in Figure 2 is to start with an image and automatically segment it into its relevant components. Our initial segmentation strategy most closely follows [4]. Using a boundary classifier such as Ilastik [6], each pixel in the image is classified to be either boundary or not boundary. This produces a boundary map. From this boundary map, a watershed algorithm is performed so that connected non-boundary pixels form basins called watershed regions or superpixels. (For the remainder of the paper, we will generally refer to any 2/3D watershed region as a superpixel.) These superpixels are considered atomic elements that will be the building blocks for the final segmentation. A Region Adjacency Graph (RAG) can be extracted from the watershed where the nodes are superpixels and an edge between superpixels indicates adjacency. The superpixels can be merged together in a process called agglomeration. Without ground truth, the agglomeration routine usually deploys some heuristic to determine when to stop. One straightforward strategy involves using the mean boundary value between two superpixels as the criterion for determining whether a merger should be performed or not. In [4], the authors describe an approach where the edges between the superpixels in the RAG are classified into a true edge or a false edge. A false edge indicates an edge that can be removed.

The quality of the segmentation algorithm is determined by measuring its similarity to ground truth. In this paper, we primarily use the Rand Index as described in [7], [2], [3]. There are other similarity measures, such as warping error [8], which are likewise effective in measuring similarity. We chose the Rand Index primarily because of its conceptual simplicity, linear-time computation, and because it is a foundation for the algorithms introduced later. (The straightforward implementation of the Rand index takes quadratic time. This algorithm is reduced to linear complexity by assigning each pixel in both images to a unique bin determined by the segmentation partition it belongs to. Pair-wise similarity is determined by simply determining the cardinality of disagreements of image A compared to image B's partitions and image B compared to image A's partitions.) In the following paragraphs, a brief description of the Rand Index (RI) [7] is provided.

The Rand index is a measure of the pairs of pixels that agree in a segmentation partition between the segmentation being assessed and ground truth. More formally the Rand index is given by the following formula:
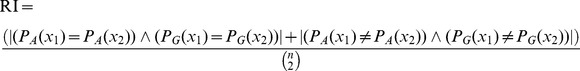

*P_A_(x)* and *P_G_(x)* are functions that respectively indicate which partition in image *A* being analyzed and ground truth *G* that a pixel, *x*, belongs. For example, P_A_(X_1_) = P_A_(X_2_) means that pixel X_1_ and X_2_ are in the same partition in image A. *n* is the number of pixels in the image. If a pixel is in two different partitions or in the same partition in both images, the index increases. The absolute value indicates a summation over all pixel pairs in the image.

The Rand index is limited in usefulness because it is not normalized. A random segmentation compared to ground truth will receive different Rand index values depending on the granularity of the segments in the ground truth. There is also a tendency for images with several small segments to receive a very high Rand index even for a bad segmentation. The adjusted Rand index (ARI) [9] is defined as follows:
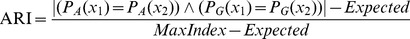
(2)Where *MaxIndex* and *Expected* are normalization factors defined as follows:
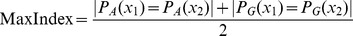
(3)

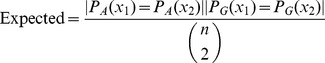
(4)
*MaxIndex* represents the average granularity between the segmentations of the ground truth and the comparison image *A*. This gives the maximum possible similarity between image segmentations. *Expected* represents an average correspondence between image segmentations. The resulting ARI is normalized so the maximum value is *1* (or *100%*) and the expected score given by comparing two random partitions is *0*.

### 2 EM Reconstruction

After automatically segmenting an image, there will often be discrepancies with the ground truth. When discrepancies occur, some applications may require manual verification and correction of the segmentation. In this section, we explore the application of reconstructing neuronal cell shapes from electron microscope (EM) images. Reconstructing neurons in EM data is an important aspect of determining neural connectivity. More motivation for this application can be found in [1].

We will briefly describe the procedure in [1] that is used to motivate the methodology introduced in this paper. First, a small specimen is prepared and sliced into thin sections. These slices are imaged one at a time using an electron microscope. The resulting EM images are transformed and aligned to form an anisotropic image volume. It is anisotropic since the resolution of EM is typically much higher than the thickness of the images slices, even though they are sliced as thinly as possible. (Typical values are 4 nm/pixel in X and Y, and 50 nm in Z). 2D segmentation and agglomeration are performed on each section and these segments are aligned to form a 3D reconstructed body. These reconstructions typically are small subsets of the entire neuron and must be merged together manually. As reported in [1], reconstructions of small parts of a fly (*Drosophila*) optic system can take several months. It is our hope that the techniques here will significantly reduce this manual reconstruction bottleneck.

### Optimizing Segmentation Turn-Around Time

In this section, we introduce an optimization criterion to motivate the algorithms in this paper. The goal is to minimize segmentation turnaround time while maintaining a level of similarity with the accepted ground truth. We define the following objective:
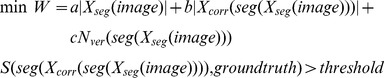
(5)where the variables *W* refers to work/turnaround time, *threshold* is minimum quality for the final segmentation, and *seg* refers to a function whose input is a set of segmentation operations, *X_seg_*, and output is a segmentation. *S(seg(X_corr_(seg(X_seg_(image))))*, *groundtruth)* is the similarity of the final segmentation to ground truth. Conceptually, S can be the Rand index, warping error, or any appropriate metric for evaluating the quality of the segmentation. In this work, variants of the Rand index will be used.

*a*, *b*, and *c* represent weighting terms. *|X_seg_(image)|* refers to the number of operations needed to perform automatic segmentation, where *a* is a small constant due to computing speed. In addition, because *a|X_seg_(image)|* is generally substantially smaller than manual segmentation (and compute resources tend to be relatively inexpensive), we can drop this term from the formulation. *X_corr_(seg(X_seg_(image)))* refers to the set of operations required to manipulate/correct an incorrect segmentation. *b|X_corr_(seg(X_seg_(image)))|* corresponds to the nuisance metric which indicates the amount of work that someone needs to correct a segmentation. For the sake of simplicity, our model assumes that *X_corr_* is composed of identically weighted operations. We will later explain how this is a valid assumption. *N_ver_(seg(X_seg_(image)))* is a function of the volume of image data and reflects the amount visual inspection performed (without modification).

We now motivate an even simpler formulation where *|X_corr_|* and *N_ver_* are combined into one term. To verify the correctness of an image, the neighborhood around each pixel is examined to check whether a neighboring pixel is a member of the same segmentation, or, similarly, whether any pixel constitutes a boundary. This operation is simplified greatly when pixels are combined into atomic superpixels, where a boundary between two superpixels can be viewed as a single edge. The manual evaluation of such an image can now be formulated as a set of yes/no questions: for each pair of adjacent pixels/superpixels, are they connected? In the worst-case scenario where all the pixels/superpixels are disconnected, the number of questions posed would be equal to the number of edges. When connections exist, this number can be much smaller due to transitive inference. Our new objective is:
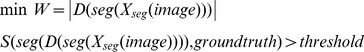
(6)where *D* now represents the set of decisions performed on the initial automatic segmentation. In other words, work is now defined as the number of these decisions. The quality of the resulting segmentation increases monotonically as more decisions are made.

A major assumption of the above formulation is that the manual segmentation can be decomposed into a set of equally hard yes/no decisions. We have observed in previous EM reconstruction efforts that this is essentially true.

Decisions involving boundaries with sufficient evidence or size are equally simple to make. However, the last 1–2 percent of small processes in the segmentation is difficult to manually correct due to the limits of EM resolution. Intuitively, this suggests that above a certain threshold of boundary evidence, the difficulty of deciding how to correct part of segmentation is constant for a given expert and image type.

In the following sections, we will propose algorithms for finding the optimal *D*, *D_opt_*, which satisfies this formula. In addition, we will introduce a similarity measure that does not depend on ground truth that is generally not available.

## Segmentation Uncertainty

Agglomeration requires the merger of adjacent superpixels. This can be done automatically or manually. In this section, we expose the uncertainty involved with agglomeration. The infrastructure discussed in this section is used in the next section, where we introduce a measure to effectively exploit uncertainty.

### 1 Probabilistic Segmentation Graph

After a watershed is created from an image, we create a Region Adjacency Graph (RAG) as defined in Section 2, which indicates the adjacency between superpixels. We then determine a weight for each edge in the RAG. This weight is determined by calculating several features (such as minimum edge pixel value, mean boundary, edge size, etc [4]). Each edge is classified as either true or false using a random forest classifier, as in [4]. Unlike [4], we do not directly use the true/false classification to label the edge, rather, we use the uncertainty in the prediction to be an edge weight. In practice, we can use any classification algorithm that can annotate a probability of an edge being a true edge.

The resulting RAG with probabilistic edge weights constitutes a *probabilistic segmentation graph*. We make some assumptions about the segmentation graph. 1) Each edge probability is taken as being independent evidence. 2) The size of the edge boundary (used as a feature) implicitly influences the edge probability through the classification. As a result, small faces between adjacent superpixels will probably lead to higher uncertainty. However, the impact of deciding which superpixels should be merged or not depends on both confidence and the size of the superpixel.

The approaches outlined in this paper rely on, to some degree, the quality of the segmentation graph. If the probabilities returned by the edge classification greatly differ from actual ground truth, our estimates and algorithms will be off. In our experiments, we show some results to suggest that the quality of the segmentation graph does significantly impact prediction. We also show that our current methods show considerable improvement over random prioritization. Since the uncertainties produced by the random forest classification are out-of-bag and random forests behave reasonably well in the presence of small variations in input, we believe the uncertainty on the training volume to be a reasonable representation of the actual accuracy exhibited on the test volume.

Without any further infrastructure, the probabilistic segmentation graph can be used to analyze the quality of segmentation and provide an ordering for manual reconstruction. When agglomerating superpixels automatically, it is not straightforward to know when to stop merging superpixels together. The probabilistic segmentation graph can indicate whether an edge should or should not be removed. If no manual verification is possible, an agglomeration algorithm might use a threshold of 50% on the edge probability, so that edges with probability greater than 50% (indicating confidence in connectivity) are removed. Alternatively, if manual reconstruction is performed, the segmentation might be conservatively refined so that only edges with connection probability greater than 90%, for example, are eliminated. In a similar manner, this probability can indicate which edges should be examined for decisions during manual reconstruction. Potential prioritization strategies involve ignoring edges with edge probabilities above and below a certain threshold. For example, the remaining edges might be ordered so that the most uncertain edges are examined first, so that the segmentation quality can be improved quickly.

**Figure 3:**
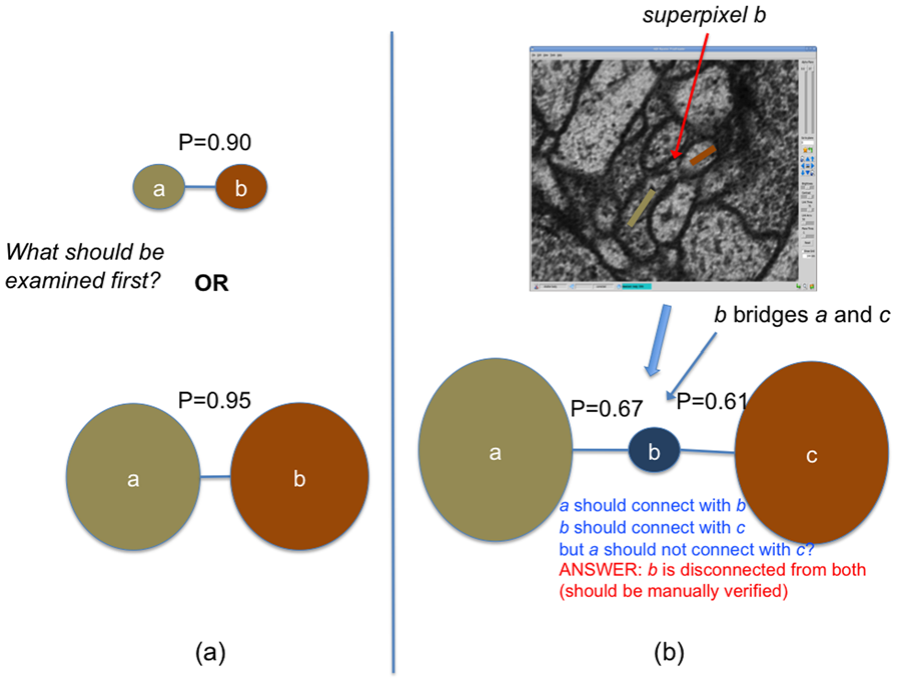
Challenges in using edge probability to determine agglomeration strategies and manual reconstruction priority. (a) Examining edges solely based on edge probability is not always ideal since it does not account of the size of the connected superpixels. (b) a and c should not be connected even though both local connections a–b and b–c indicate a connection.

However, edge probabilities do not indicate which decisions made during manual reconstruction are most topologically significant. Consider Figure 3a where a connection between two small superpixels could be examined before a more certain connection between larger superpixels. In the next two sections, we will introduce new measures for determining which edge is more important to examine first. In addition, local edge probability can be misleading when agglomerating superpixels. In Figure 3b, there are two superpixels connected by another superpixel, *b*, which acts as a bridge. Both edges connected to *b* indicate a connection with probability greater than 50%. If the agglomeration algorithm eliminates one edge, the other edge might also be eliminated since it is also greater than 50%. However, the initial watershed indicates that the probability that *a* and *c* are connected is less than 50%. In this manner, it is possible for large topological errors to occur after agglomeration. Therefore, it is important to consider how likely two superpixels are connected globally without only examining the local connections. We explain the strategy to compute this information in the following. This global connectivity will then be leveraged in the next section.

### 2 Pair-wise Connection Strength

To enable a more global assessment of connectivity, we compute connection strength between every pair of superpixels in the probabilistic segmentation graph. For instance, if the edge probability between *a* and *b* is 5%, the connection strength can be much greater if there is another path through the graph with a higher connection strength. Therefore, to find the absolute connection strength between *a* and *b*, we must compute the connection probability for every path between *a* and *b*. Computing this connection probability is daunting for two reasons: 1) for *n* superpixels there are n^2^ pairs and 2) there can be an exponential number of paths between two superpixels.

To greatly simplify this calculation, we approximate the connection between two superpixels by examining the strongest connection path between two nodes. With this simplification, Dijkstra's algorithm [10] can be applied. The algorithm described below finds the shortest path from a source superpixel A to all other nodes in the RAG:

1. Start with a given superpixel, A, where all other superpixels are connected with 0% probability.
2. Add A to the heap and set the current connection strength to 1.
3. Grab the most connected superpixel on the heap and set the current connection strength
  1. Add to pair-wise connection table with connection strength
  2. Examine all edges connected to the superpixel
  3. For each unexamined superpixel, multiply the edge connection probability to the current connection strength and add to the heap

This variant of Dijkstra's algorithm, as with the classic version the Dijkstra's algorithm [10], has worst-case complexity of O(|E|+|V|log|V|) using a Fibonacci heap, and is quite fast for any given node pair However, computing the distance between all N∧2 pairs of nodes in the RAG can still be prohibitive (especially if a more inclusive shortest path algorithm is used – see below). However, the vast majority of potential connections are so improbable we do not need to compute their exact value. Therefore we establish a connection threshold that determines when the algorithm terminates. For the graphs we use here, this limits exploration of the graph to only a few nearest neighbors, with a concomitant decrease in execution time. For large graphs, it is also possible to split the problem up into small overlapping regions since it is unlikely that a superpixel would be connected to something distant (measured in terms of the number of edges between the superpixels).

If more accuracy is desired, more paths can be considered between any two superpixels to provide a better estimate of affinity between the nodes. In our experiments, we have noticed that considering more paths does not greatly impact our results (especially given the extra computation required) and consider only the highest probability path. For completeness, we briefly discuss computing connection strength using multiple paths in Appendix A of Text S1.

## GPR – Evaluating Segmentation in the Presence of Uncertainty

From the pair-wise connection probability, we compute a novel measure called the ***Estimated Generalized Probabilistic Rand Index (GPR)***. This measure quantifies the certainty in the probabilistic segmentation graph, where the set of pair-wise connection probabilities encodes a superposition of several different segmentation configurations with varying degrees of likelihood. GPR extends the Rand index to the situation where no ground truth is available to compare the segmentation against.

The estimated GPR introduced in the paper has some similarities to the Normalized Probabilistic Rand Index (NPR) introduced in [3]. In that work, the authors devise NPR to facilitate comparisons between different segmentations and ground truth from multiple sources. However, the target domain of our work is very different. In this paper, we derive a measure to quantify the uncertainty in a segmentation graph, in addition to having the capability of evaluating specific segmentation algorithms. While the probabilistic segmentation graph can be considered a limiting case of generalizing multiple ground truths, the mechanism for determining these probabilities is very different (a result of automatic edge classification). Our approach affords us the luxury of evaluating our algorithms with minimal ground truth allowing us to guide segmentation refinement, the dominant time bottleneck in connectomic reconstruction. On a more technical note, the normalization of GPR is defined to closely follow the Adjusted Rand Index [9], so that we can account for differences in segment granularity between images. We introduce a novel and efficient formulation to enable this normalization in the following paragraphs.

We now define the unnormalized GPR (U-GPR) in a manner analogous to Rand index in Equation 1:
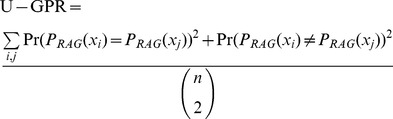
(7)
*X_i_* refers to individual pixels; however, the formulation above can be simplified by summing over each superpixel (weighting by the size of the superpixel accordingly). The summations will now be explicitly shown (unlike in Equation 1) to make the formulations clearer. *Pr()* refers to the probability of connection between the two superpixels. P_RAG_(x_i_) indicates the partition id that x_i_ belongs to with respect to the probabilistic segmentation graph or RAG. The formulation can be considered as a comparison of the probabilistic segmentation graph to itself. If there is complete certainty, then there will be absolute agreement in connectivity.

As with the Rand index, the U-GPR, by itself, does not give meaningful results since the value returned varies depending on the granularity of the underlying ground truth (in this case embedded in the probability). For images with hundreds of distinct neurites, the U-GPR will likely be close to 1. GPR is the normalized version of U-GPR and is analogous to the adjusted Rand index in Equation 2:
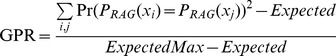
(8)ExpectedMax corresponds to the expected granularity of the probabilistic segmentation graph. This can be visualized as randomly picking two configurations of the segmentation graph and computing the maximum correspondence possible as done for the adjusted Rand index. When this is averaged over all the possible segmentation configurations in the probabilistic segmentation graph (weighted by their likelihood), the result is simply the expected granularity of the graph given by:
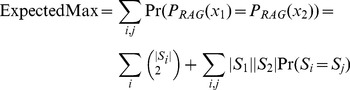
(9)Where *S* and *|S|* correspond to a superpixel and the number of pixels in the superpixel respectively. This normalization determines the expected granularity of segmentation and that provides an estimate of the highest likely correspondence to the uncertainty graph. Notice that the expected granularity of the probabilistic segmentation graph consists of the granularity of the superpixels added with the number of pairs between two superpixels multiplied by the likelihood that they are connected. Because the pair-wise connection probability calculated already account (or approximate) for dependencies between the superpixels, each superpixel pair can be analyzed separately. Referring back to Equation 8, *Expected* can be defined as:
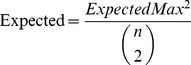
(10)Computing the GPR requires only a linear traversal of the pair-wise connections between the superpixels. In the worst case, there is quadratic number of connections in the RAG; however, in practice, the number of pair-wise connections is significantly smaller as per the discussion in the previous section. The algorithm used to calculate pair-wise probability dominates the complexity of GPR. More details of this computation can be found on publically available software at https://github.com/qedq/NeuroProof.

The GPR metric can be viewed as a measure of the normalized deviation in the probabilistic segmentation graph. When the deviation is small, there is high confidence in the underlying probabilistic graph. The ‘ground truth’ is the underlying edge probabilities and the derived pair-wise connection probabilities. As we will show in the experiments, the fidelity of the GPR depends on the quality of the edge classification. While the GPR measures the confidence in the probabilistic graph, and similarly, the edge classification, the graph is not a specific segmentation but rather a superposition of several possible configurations. GPR can be extended to compare a concrete agglomeration to the probabilistic graph. We include this formulation in Appendix B of Text S1. We will use this result to estimate the quality of an agglomeration algorithm. Ideally, the GPR would be a tight lower bound to the actual adjusted Rand index if ground truth were available.

## Manual Correction in the Presence of Uncertainty

After an automatic segmentation is produced, manual verification is necessary to assure correctness. However, manual verification requires the visual inspection of the entire segmentation. While a quality automatic segmentation reduces the number of corrections required, the turn-around time will reach a lower limit unless at least some parts of segmentation can be ignored. A straightforward strategy would be to ignore all edges with a certain level of certainty. Such a strategy does not properly distinguish between a segmentation where every edge is slightly above the threshold or where only half are. We introduce a manual verification strategy that uses a global budget, reflected by the GPR, as a means of substantially reducing the amount of work, and therefore turn-around time, required.

**Figure 4:**
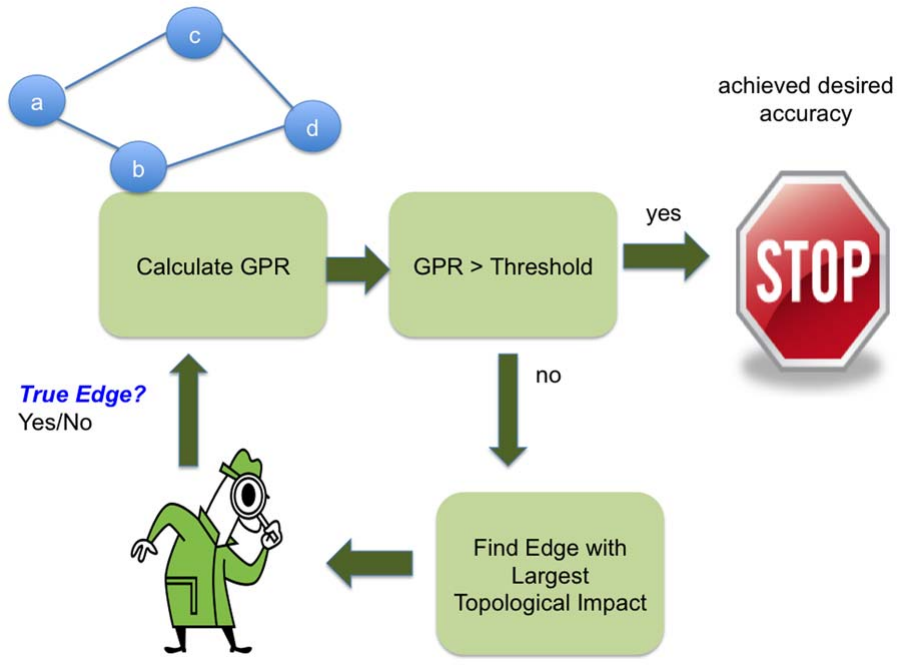
Manual verification methodology. GPR is used to both prioritize edges to be examined and terminate the manual work.

We define this objective in a similar manner to Equation 6:
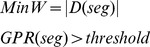
(11)where the goal is to minimize the number of decisions needed to achieve a certain GPR value. We achieve this goal using a methodology outlined in Figure 4. Starting from the probabilistic segmentation graph in the top left of the figure (edge probabilities not shown), the GPR is calculated. We then check if the GPR is greater than some cut-off threshold, such as 90%. If it is, the segmentation is considered to be at a reasonable level of confidence. If not, we find an edge in the RAG that is both uncertain and whose resolution could lead to the largest topological change, which we will discuss in detail in the next paragraph. We then assign a person to manually check whether the edge is true or false. This information is then used to update the probability for that edge in the graph by setting it to 1 (in the case of a connection) or 0 (in the case of a true edge) and the GPR can be recalculated. In practice, when a false edge is removed and two superpixels are merged, we could recompute the edge probabilities to adjacent superpixels where the boundary evidence has changed to further improve the GPR estimate. We avoid this additional step in this work.

Finding the edge with the largest topological impact is done in a greedy manner by choosing the most *impactful* edge. (Greedy order is not necessarily optimal. Finding an optimal order is complicated by runtime complexity and the indeterminism introduced by human interaction that prevents an absolute knowledge of how future decisions will be made.) We determine the most impactful edge with this measure:
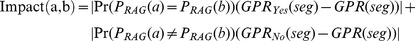
(12)This measures the impact of the edge between adjacent regions *a* and *b* (for generality, the region could consist of multiple merged superpixels). The first absolute value term is the change in GPR that occurs if a manual decision yields a false edge result (or yes decision). The likelihood of this change is given by the edge probability between the two regions (determined by the initial edge classification). The edge probability between two superpixels (or regions when the edge probability is recomputed after a merger) determines the likelihood since an actual decision is restricted to the local edge between the regions. It is not based on some measure of global connectivity. The second absolute value term determines the change in GPR due to a true edge (or no decision). If a given edge has very high or very low edge connection probability, this impact will be very small since either outcome will only minimally impact the GPR. Notice that this measure finds the absolute expected change in GPR without requiring the change to be an increase. This emphasizes decisions that make large topological changes to the graph independent of whether the immediate result is an increase in graph certainty.

Computing the impact measure in Equation 12 can also be time-consuming since it requires calculating the GPR resulting from a yes and no decision on each edge. In addition, because the pair-wise probabilities are updated after every manual decision, this ranking will change for each iteration in Figure 4. To avoid this prohibitive runtime cost, we update the impact ranking after several decisions are made with the hope that the ranking does not change significantly. Furthermore, we heuristically ignore edges whose local edge probability is above and below a certain threshold (compared to the other edges). In practice, this ranking can be continually updated with a background process improving the ranking while manual decisions are made.

Using the GPR as a guide and cut-off mechanism is a proxy for comparing the segmentation to a ground truth using a measure such as the adjusted Rand index. An initial segmentation with a low GPR might not give a score similar to an actual Rand index. However, the low score indicates inherent ambiguity in the classifier and reveals, through our impact measure, what parts of the segmentation need to be examined. As more confidence is added to probabilistic segmentation graph through manual decisions, the GPR slowly reduces into a more concrete representation of the ground truth. As we show in the next section, the number of decisions required to greatly improve the GPR number is a small fraction of the total number in the RAG.

## Materials and Methods

**Figure 5:**
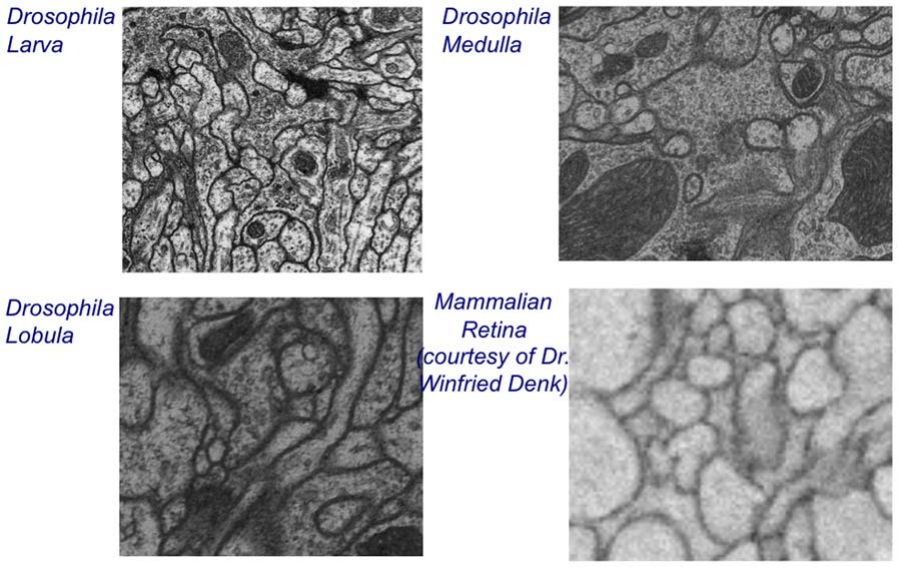
EM data samples used in the experiments.

In our experiments, we consider EM image samples from the *Drosophila* Larva, *Drosophila* Medulla, and *Drosophila* Lobula, obtained from Janelia Farm Research Campus, and mammalian Retina, courtesy of Dr. Winfried Denk (sample prepared in [11]), as shown in Figure 5. Training is performed on one 2D EM slice from each sample. Training entails boundary labeling and edge classification, which is determined by comparing to a ground truth segmentation of the sample. Testing is performed on another 2D EM slice from each sample located near the training slice.

A boundary classifier is created from each image in the training set using Ilastik [6]. One classifier is applied to both training and testing images for each sample to produce a boundary map from which a watershed is created. In practice, each sample trained in Ilastik only requires a few hundred ground-truth label points to produce a high-quality boundary classification. Ground truth is then created from the watershed volume. Although our methodology assumes each superpixel in the watershed is atomic, it is possible for the ground truth to violate this assumption. This happens infrequently and does not appreciably affect the results. Using the training image ground truth, we classify the edges between the superpixels. We derive the following features over the boundary probability map to be used in edge classification: 1) mean pixel value, 2) minimum boundary intensity, 3) maximum boundary intensity, 4) size of both nodes, 5) difference of average intensity between the interior of the nodes, 6) multiplication of both nodes' difference in average intensity between the edge and interior, 7) max between the two nodes' edge intensity normalized by their size, and 8) the size of the edge. The classification is computed using random forest as in the work in [4] using the default settings in VIGRA (http://hci.iwr.uni-heidelberg.de/vigra/).The classification is then applied to the test image watersheds to produce connection probabilities on each superpixel edge. This is the starting point for the following experiments.

## Empirical Results

We now demonstrate the effectiveness of GPR to guide manual segmentation refinement. First, we show that GPR can be used as a cut-off mechanism to determine when manual reconstruction can safely terminate. Second, we explore how the GPR relates to the actual adjusted Rand index against ground truth. Finally, we examine various performance details of our methodology in terms of the runtime, consistency, and quality.

### 1 Reducing Manual Reconstruction Effort

Table 1 reveals the reduction of manual reconstruction effort (number of edges examined) to achieve a threshold of segmentation quality without examining some of the edges. The second column gives the GPR before any manual examination of the edges. The next three columns, GPR, Random, and Edge Probability priority are three strategies for ordering the edges to achieve the segmentation threshold fastest. For the GPR and Random priority, the threshold is reached when the GPR is >90%. For the Edge Probability priority, the threshold is a reached when all edges with connection confidence 10–90% are examined. For this experiment, the manual reconstruction is simulated by software that labels each edge yes/no based on the ground truth.

**Table 1:** Reducing reconstruction effort through prioritization.

		Percent Effort to Proofread 90%		
EM Image	Initial GPR	GPR Priority	Random Priority	Edge Probability Priority
Larva	48%	25%	78%	41%
Medulla	35%	46%	95%	67%
Lobula	41%	38%	83%	53%
Retina	84%	2%	37%	27%

GPR priority uses GPR to rank the most significant edges. Random priority orders the edges randomly. Edge probability priority ranks the edges based on the connection probability determined by the classifier.

The table shows that the GPR Priority consistently considers fewer edges to reach its threshold as compared to the Random and Edge Probability Priority. The great improvement compared to Random Priority indicates that even with the same stopping metric, the decision order can greatly effect how quickly the GPR increases. The Edge Probability Priority indicates there are more edges with connection certainties between 10 and 90 percent than edges needed to reach a 90% GPR using the GPR Priority. The increase in work required for Edge Probability suggests that many edges whose connection are not topologically significant enough to be examined using the GPR Priority. In particular, note that in the Retina only 2% of the edges needed to be manually verified to achieve 90% confidence in the segmentation, compared to 27% needed for Edge Probability Priority.

### 2 Accuracy of GPR Metric

Manual reconstruction generates yes/no decisions on uncertain edges. When this information is re-incorporated in the connection graph, we run an agglomeration algorithm that speculatively merges superpixels to ideally generate a segmentation closer to ground truth. Future work will involve directly using the GPR metric to guide agglomeration. For these experiments, we perform agglomeration by merging superpixels with the largest mean boundary value agglomeration. We report the highest adjusted Rand index for all mean boundary thresholds. In Table 1, the highest adjusted Rand index for all samples after the manual decisions with every priority strategy was greater than 90%. This indicates, at least for these examples, that the predicted similarity with the ground truth is a lower bound of the actual similarity with ground truth.

**Figure 6:**
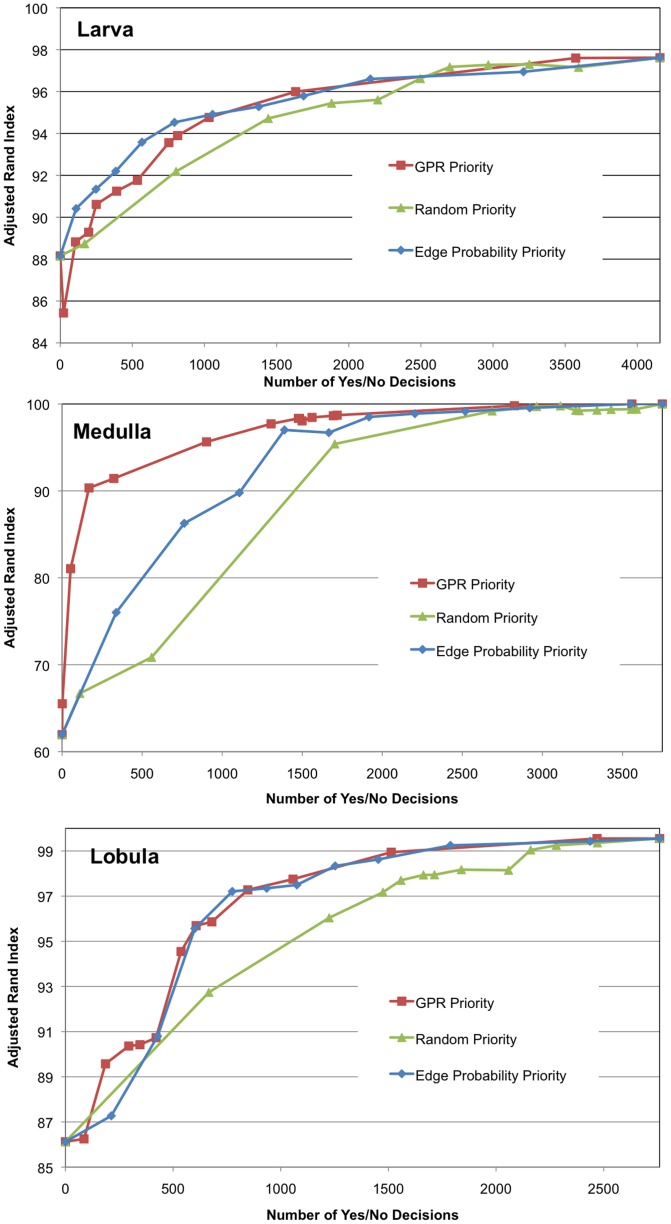
Adjusted Rand Index as a function of manual reconstruction effort. Effort is determined by the number of yes/no decisions. GPR Priority dominates Random Priority in each sample.

Figure 6 shows that GPR-based priority achieves better similarity with ground truth with fewer decisions (with respect to mean boundary agglomeration) than random priority. Edge probability and GPR-based priority perform similarly on the Larva and Lobula but the GPR-based priority greatly outperforms edge probability for the Medulla. In other words, these results indicate that by using GPR-based priority, greater similarity with ground truth can be achieved with similar amount of effort to other techniques. Data on the mammalian retina is excluded from the analysis since the initial similarity with ground truth is very high (>95%).

**Figure 7:**
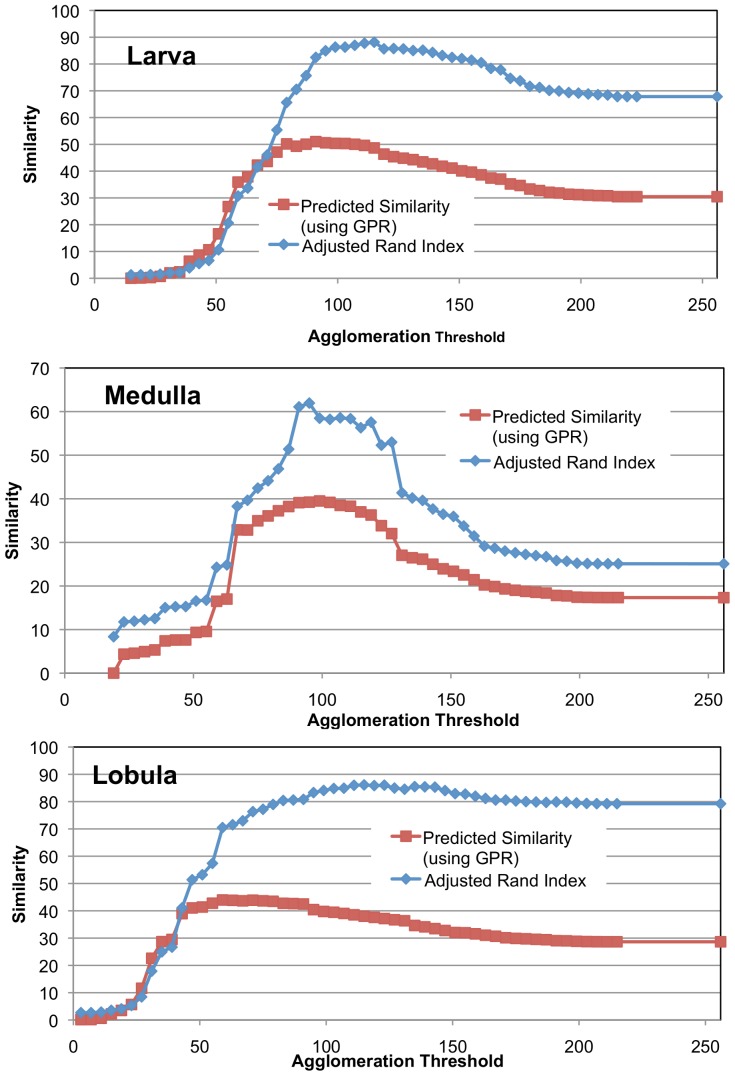
Shows the predictiveness of GPR similarity measure compared to the actual adjusted Rand index computed with an expert-created ground truth. The y-axis gives similarity as a function of different agglomeration thresholds. The agglomeration is based on the mean intensity of the pixel-wise boundary prediction.

We also show the quality of GPR by noting how well it predicts the similarity of a segmentation to ground truth. While automatically agglomerating superpixels, the best stopping threshold is unclear without ground truth. We compare the probabilistic graph to each segmentation with the anticipation that the highest degree of correspondence will occur at a threshold where the actual similarity to ground truth is greatest. While it is possible to selectively choose edges that correspond with ground truth but refute the initial evidence, we hope that the combination of a quality probabilistic segmentation graph and an agglomeration algorithm that is not an explicit adversary of the measure leads to good prediction of similarity. Figure 7 shows how well the GPR-based metric models the actual adjusted Rand index for different mean agglomeration thresholds. Threshold 0 means that every superpixel boundary pixel is considered a connection. Threshold 256 means that every superpixels boundary pixel is not a connection. Notice that in the Medulla the GPR-based prediction correspondence closely to the actual ground truth suggesting an optimal agglomeration threshold around 100 (compared to the straightforward 128 stopping point for mean agglomeration). The threshold predicted for the Larva and Lobula is not quite optimal, but the predicted curve tracks closely with the actual curve and is an under-estimate.

**Figure 8:**
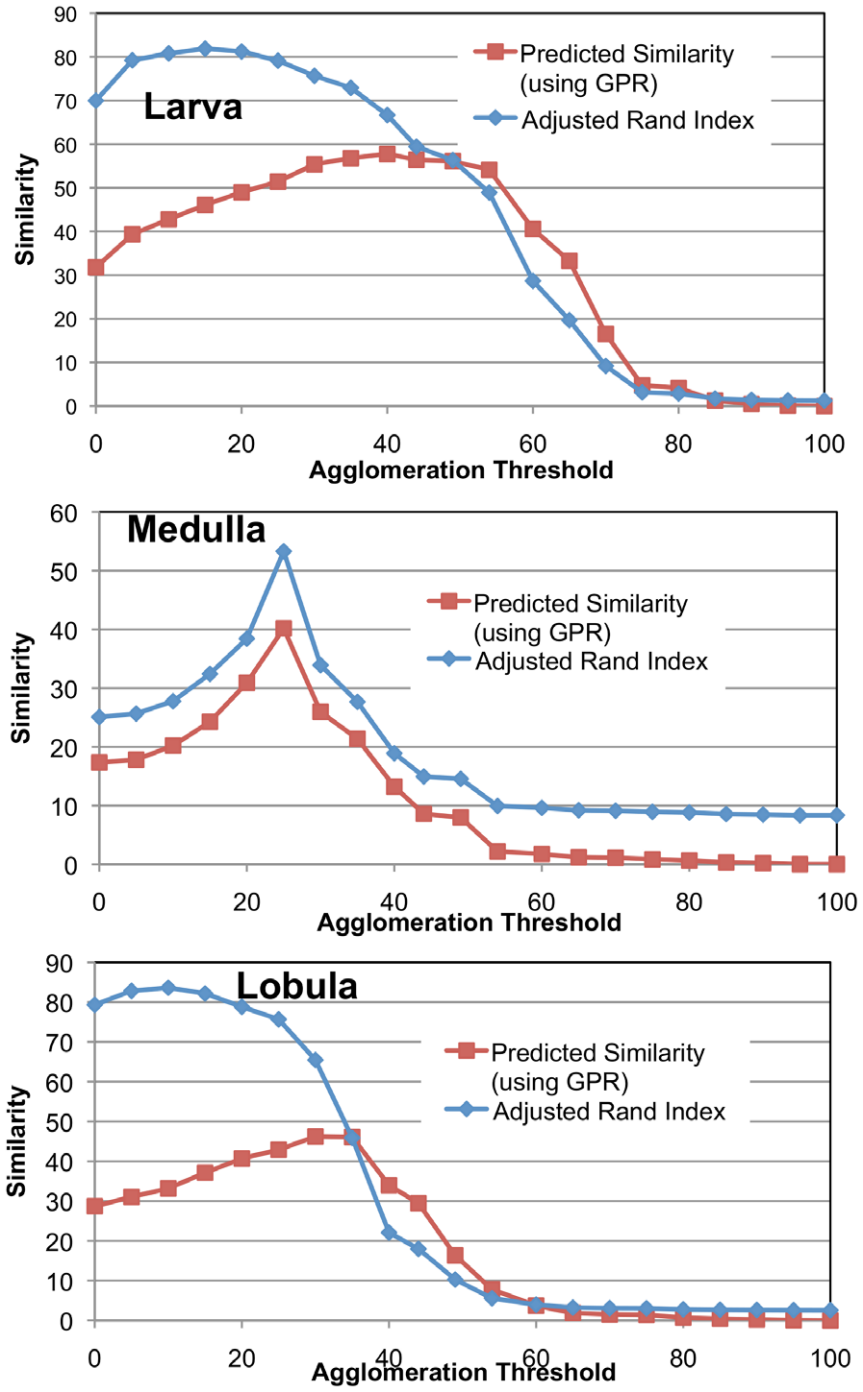
Show the predictiveness of GPR similarity measure compared to the actual adjusted Rand index. Unlike Figure 7, the agglomeration is based on the edge connection probability, not the mean intensity of the boundary.

In Figure 8, we show the effectiveness of predicting the similarity of a segmentation to ground truth using a different agglomeration strategy. This agglomeration eliminates all edges with X% certainty of not being an edge. As with the previous example, our similarity measure predicts actual similarity in the Medulla closely. In addition, the 50% threshold does not yield close to the optimal solution, whereas the GPR-based similarity measure is much closer to optimal.

### 3 Performance Details of GPR

This section will first discuss the impact of the edge classification on the quality of the GPR result. We will then discuss the implication of considering more than one path in the GPR calculation.

The GPR analysis is dependent on the probability generated by the random forest. We first examined the sensitivity of the GPR metric on small changes to the probabilistic segmentation graph. Performing 5 different edge classifications obtained from different random starting seeds on the Larva sample, we see only a small variation in the GPR and GPR-based effort numbers reported in Table 1 of less than 0.5% and 1% respectively.

**Figure 9:**
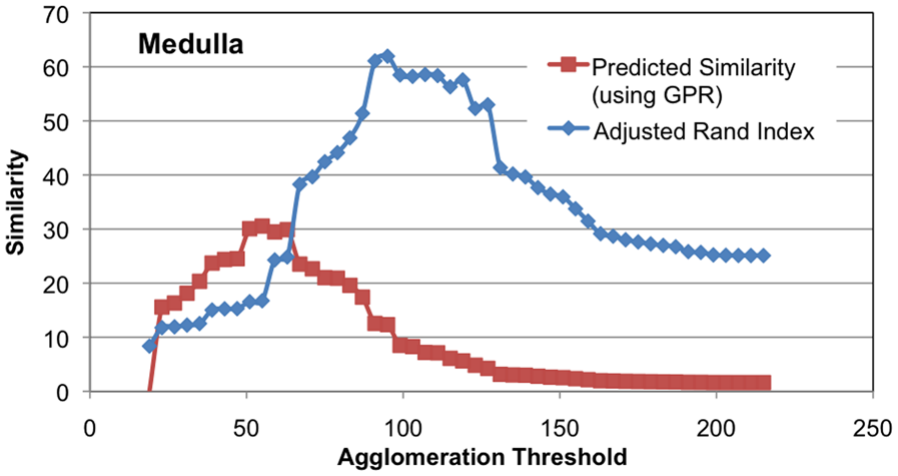
The poor predictiveness of GPR similarity measure compared to the actual adjusted Rand index. While the agglomeration is based on mean intensity as in Figure 7, the quality of the edge probabilities is degraded by only using mean intensity as a feature for computing edge probability, thus leading to poor correspondence between GPR and the adjusted Rand index.

In future work, we want to develop a measure to indicate whether the probabilities obtained through classification are good. However, we have observed that choosing a very simple set of edge features for the random forest classifier leads to poor results. We experimented with a simple classification routine that only considered the mean value along an edge. With this classification, the larva failed to achieve an adjusted Rand index of 90% despite reaching GPR of 90% suggesting that many edges were incorrectly being ignored due to an incorrect high degree of certainty. The impact of this classifier is more profound when examining the predictiveness of the GPR measure of the adjusted Rand index. In Figure 7, the optimal threshold was accurately determined for the Medulla sample using GPR. With the simpler classifier, the quality of the prediction is seriously degraded as evidenced in Figure 9.

**Figure 10:**
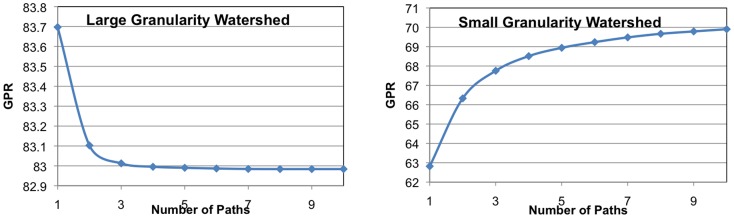
The quality of GPR approximation as a function of the number of paths considered. Note that small magnitude difference between one path and the maximum number of paths.

Calculating the GPR for each sample takes on the order of seconds using only one path, for the sample images, as it is just a variation of Dijkstra's algorithm. The speed of this computation makes it a more attractive approach than the extra bookkeeping required for analyzing multiple paths. One would expect that for a segmentation that has few false splits that one path would be sufficient since there would be fewer connecting paths. Is this the case for a more fragmented watershed? In Figure 10, we calculate the GPR using different numbers of paths for two versions of the Retina sample (chosen due to its small size): the original watershed and a more fragmented version of the watershed. In both cases, the results indicate that the GPR changes a small amount when using a more accurate algorithm of more paths. For this example, when the watershed is less fragmented, the GPR approximation is an over-estimate and quickly converges to the actual GPR. When the watershed is more fragmented, the GPR approximation in an under-estimate and also quickly converges to the actual GPR. Furthermore, the magnitude of one-path approximation error is larger for the sample with the fine-granularity watershed. However, even in this case the change in GPR is only about 7%. If the one-path approximation becomes more accurate when it is higher, each refinement of the probabilistic segmentation graph due a manual decision should produce a more accurate approximation.

## Discussion

In this work, we examine applications in image segmentation where the quality of segmentation requires manual verification. This is particularly prevalent in domains involving classification of neuronal cells and connectomics from EM data. Reconstructing these circuits, even with an initial automatic segmentation, can take months to years. In this domain, we introduce a strategy to minimize the amount of manual reconstruction effort while achieving a high quality in the refined segmentation. To this end, we propose a novel similarity metric, GPR, which determines segmentation quality from probabilities rather than requiring initial ground truth. By prioritizing which part of the image to manually verify first using GPR, we show that our segmentation converges to a high-level of similarity much more quickly than alternative prioritization strategies. Furthermore, we achieve significant reduction in manual reconstruction effort to achieve a high-level of segmentation quality. Finally, the GPR measure can be deployed as a standalone mechanism to evaluate the quality of a segmentation algorithm in the absence of a gold standard.

There are many opportunities to further improve our approach. Currently, our boundary and edge classification strive to minimize pixel/edge-wise error. Developing classifiers that minimize probabilistic uncertainty could lead to a better probabilistic segmentation graph and, consequently, a more accurate GPR. As observed in Figure 9, inadequate training data or a simple feature set can lead to less edge certainty and/or reduced generalizability, which can degrade estimation. Furthermore, an agglomeration could be created that directly leverages GPR, which should outperform mean boundary-based agglomeration. Finally, we are still exploring optimal ways of using GPR to generate a more optimal manual decision order.

## Supporting Information

Text S1 **Appendices.** These appendices go into more detail on algorithms supporting the main theory in this paper. (Appendix A) Describes how to compute the affinity between two nodes with multiple, non-independent paths. (Appendix B) Describes how to compute the similarity between a segmentation and a probabilistic segmentation graph.
(DOC)
