# Supplementary material for: Minimizing Manual Image Segmentation Turn-Around Time for Neuronal Reconstruction by Embracing Uncertainty
Source: PLoS One. 2012 Sep 21;7(9):e44448. doi: 10.1371/journal.pone.0044448 (PMC3448604; doi:10.1371/journal.pone.0044448)
Supplement: Text S1 — Appendices. These appendices go into more detail on algorithms supporting the main theory in this paper. (Appendix A) Describes how to compute the affinity between two nodes with multiple, non-independent paths. (Appendix B) Describes how to compute the similarity between a segmentation and a probabilistic segmentation graph. (DOC) [file pone.0044448.s001.doc]

**Text S1 Appendices**

These appendices go into more detail on algorithms supporting the main theory in this paper.

(Appendix A) Describes how to compute the affinity between two nodes with multiple, non-independent paths.

(Appendix B) Describes how to compute the similarity between a segmentation and a probabilistic segmentation graph.

**Appendix A: Computing Connection Strength between Nodes with Multiple, Overlapping Paths**

As described before, a simple application of Dijkstra’s algorithm allows us to find the highest affinity path between two superpixel nodes. To improve the estimate of affinity between two nodes, one should ideally consider all paths. A simplifying assumption that we examined was to choose the K-most significant paths between two superpixel nodes. This is only an estimate as it is possible for a less significant path to be disjoint from the other paths and thus contribute more to the affinity between nodes. The details of this computation are not given here. In practice, by keeping track of previous shortest paths, Dijkstra’s algorithm can be extended to produce these paths. Extra bookkeeping is required to avoid cycles.

Given K paths, we now focus on how an affinity is computed between a pair of nodes. We reduce the problem of finding connection affinity as finding the probability that every path, *L_i_*, between two nodes are not connected (*Pr(L_i_^c^)*):

(13)

The final line indicates a recurrence relation that is straightforward to implement. Pr(L_k_) is simply the probability that all edges in a path are a false edge, a condition necessary for both nodes to be connected through this path. The probability conditioned on L_k_ simply requires that those edges (if reused) be set to a connection probability of 1.

**Appendix B: Estimating Similarity Between An Uncertainty Graph and Concrete Segmentation**

Computing a concrete estimated GPR (comparison with an actual segmentation) shares some similarities to the work in [10,11] with the big exceptions being that the uncertainty graph is a novel derivation of a machine-determined edge uncertainties and the normalization mirrors the adjusted Rand Index [8]. The next three equations follow from Equations 8, 9, 10:

Concrete-GPR = (14)

CExpectedMax = (15)

CExpected = (16)

In Equation 14, a similarity between a segmentation and the uncertainty graph is determined by multiplying the connection probability between each pixel that is assigned to the same partition in the segmentation (given by I(P_SEG_(x_i_) = P_SEG_(x_i_)). The ExpectedMax and Expected values defined by Equations 9 and 10 incorporate the granularity of the concrete segmentation to produce Equations 15 and 16.
